# Supplementary material for: Decitabine-Mediated Upregulation of CSPG4 in Ovarian Carcinoma Cells Enables Targeting by CSPG4-Specific CAR-T Cells
Source: Cancers (Basel). 2022 Oct 14;14(20):5033. doi: 10.3390/cancers14205033 (PMC9599610; doi:10.3390/cancers14205033)
Supplement: Supplementary file 1 [file cancers-14-05033-s001.zip › Supplemental Figure S1.pdf]

Fig. S1

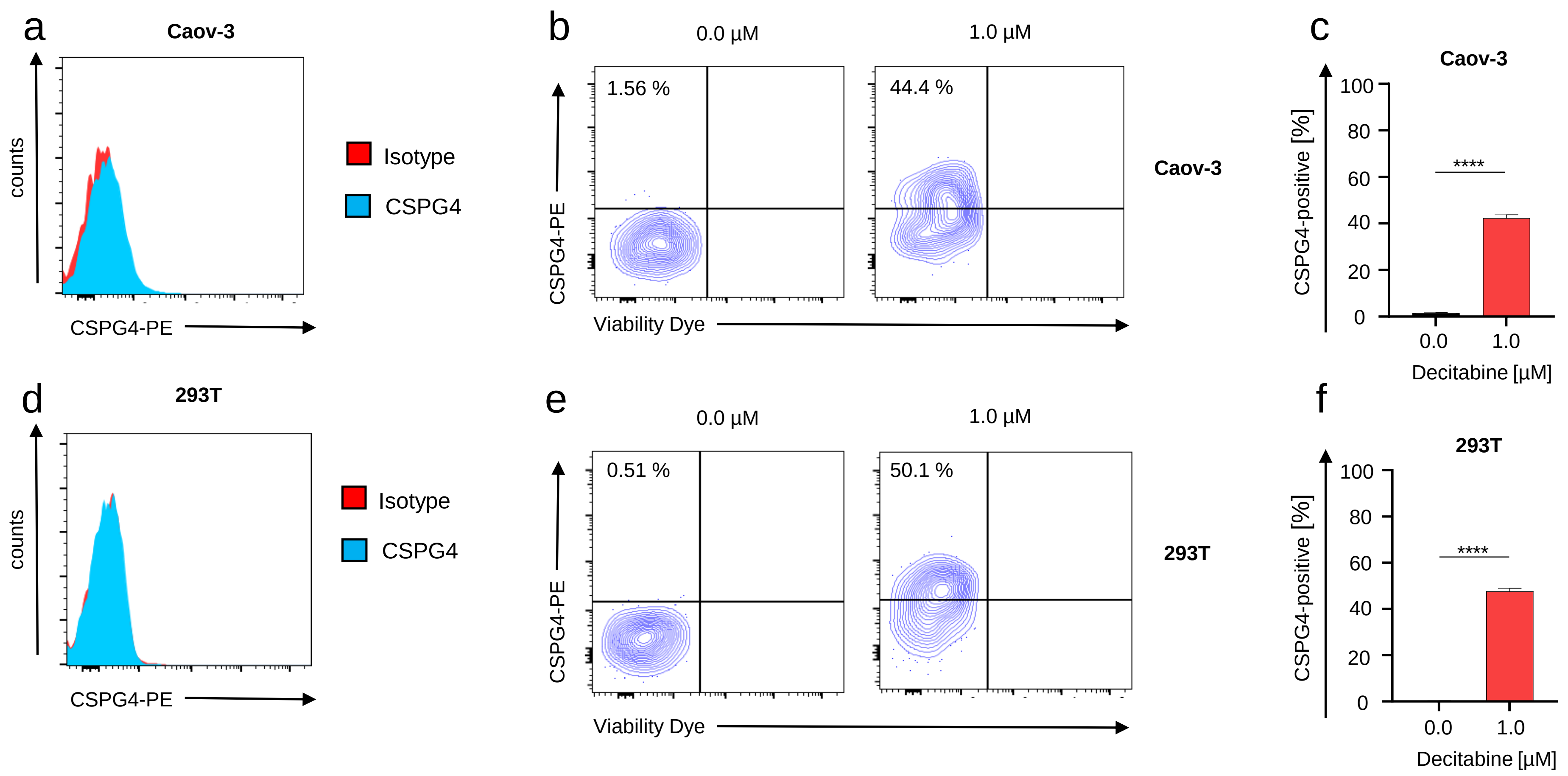

Figure S1. Decitabine-mediated CSPG4 upregulation in Caov-3 cells and 293T cells**.** **(a)** CSPG4-staining of Caov-3 ovarian cancer cells using a PE-labeled anti-CSPG4 antibody and a corresponding isotype control. One representative run out of three experiments is depicted. **(b)** Contour plots showing CSPG4-staining of Caov-3 ovarian cancer cells six days after daily addition of Decitabine at 1 µM. One representative run out of three experiments is depicted. **(c)** CSPG4-expression in Caov-3 ovarian cancer cells six days after daily addition of PBS or Decitabine at 1 µM. Data represent geometric means ± SEM of three runs, p values were calculated by Student´s t test, **** indicates $p \leq 0.0001$. **(d)** CSPG4-staining of 293T cells using a PE-labeled anti-CSPG4 antibody and a corresponding isotype control. One representative run out of three experiments is depicted. **(e)** Contour plots showing CSPG4-staining of 293T cells six days after daily addition of PBS or Decitabine at 1 µM. One representative run out of three experiments is depicted. **(f)** CSPG4-expression in 293T cells six days after daily addition of Decitabine at 1 µM. Data represent geometric means ± SEM of three runs, p values were calculated by Student´s t test, **** indicates $p \leq 0.0001$.
