# Supplementary material for: Decitabine-Mediated Upregulation of CSPG4 in Ovarian Carcinoma Cells Enables Targeting by CSPG4-Specific CAR-T Cells
Source: Cancers (Basel). 2022 Oct 14;14(20):5033. doi: 10.3390/cancers14205033 (PMC9599610; doi:10.3390/cancers14205033)
Supplement: Supplementary file 1 [file cancers-14-05033-s001.zip › Supplemental Figure S2.pdf]

Fig. S2

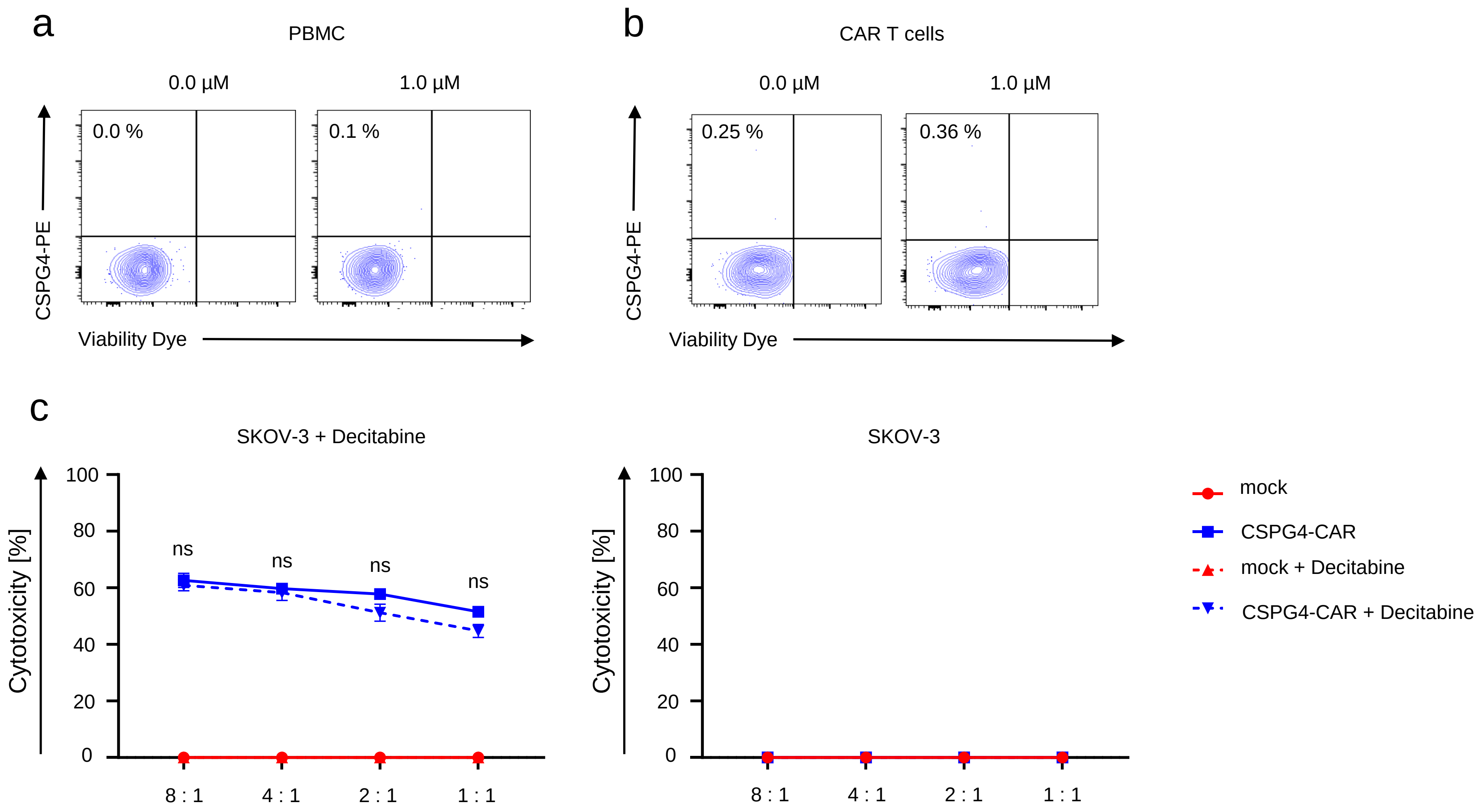

Figure S2. CSPG4 expression in PBMC and CAR T cells after Decitabine treatment**.** **(a)** Contour plots showing CSPG4-staining of PBMC days after daily addition of PBS or Decitabine at 1 µM. One representative donor out of three experiments is depicted. **(b)** Contour plots showing CSPG4-staining of CSPG4-specific CAR T cells generated via mRNA-electroporation six days after daily addition of PBS or Decitabine at 1 µM. One representative out of three experiments is depicted. **(c)** Cytotoxicity of CSPG4-CAR-T cells and mock T cells upon a 48h co-culture with SKOV-3 cells either treated with PBS or Decitabine for six days was assessed at the indicated effector to target ratios via an XTT-based colorimetric assay. Mock T cells (Red lines) and CSPG4-CAR-T cells (Blue lines) were either maintained at 1 µM Decitabine following electroporation until completion of the killing assay (dotted lines) or were maintained in regular T cell medium after electroporation and throughout the killing assay (solid lines). Data represent means ± SEM of three donors, p values were calculated by Student´s t test, ns indicates not significant.
