# Supplementary material for: Decitabine-Mediated Upregulation of CSPG4 in Ovarian Carcinoma Cells Enables Targeting by CSPG4-Specific CAR-T Cells
Source: Cancers (Basel). 2022 Oct 14;14(20):5033. doi: 10.3390/cancers14205033 (PMC9599610; doi:10.3390/cancers14205033)
Supplement: Supplementary file 1 [file cancers-14-05033-s001.zip › Supplemental Figure S3.pdf]

Fig. S3

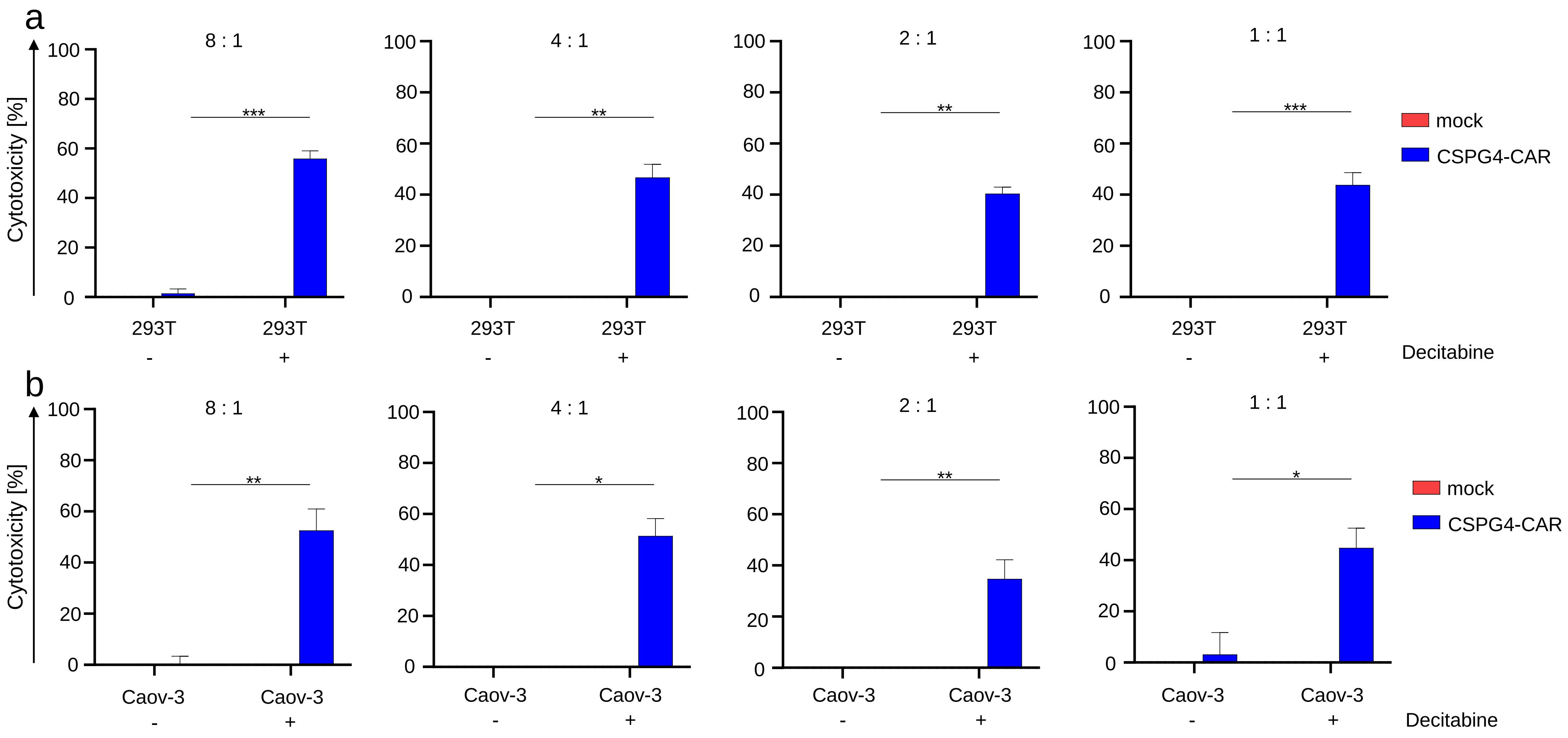

Figure S3. Lytic activity of CSPG4-CAR-T cells against 293T and Caov-3 cells. Cytotoxicity of CSPG4-CAR-T cells and mock T cells upon a 48h co-culture with 293T cells **(a)**, and Caov-3 cells **(b)** either treated with PBS (-) or Decitabine (+) was assessed at the indicated effector to target ratios via an XTT-based colorimetric assay. Data represent means ± SEM of three donors, p values were calculated by Student´s t test, *** indicates $p \leq 0.001$, ** indicates $p \leq 0.01$, and * indicates $p \leq 0.05$.
